# Application of blood rheology to develop hemolysis-free serum extraction sampling method for small-volume blood

**DOI:** 10.1016/j.plabm.2026.e00550

**Published:** 2026-07-22

**Authors:** Hideaki Isago, Baasanjav Uranbileg, Keisuke Murayama, Yuki Nakano, Yukio Kume, Yoshihiro Minagawa, Masaomi Nangaku, Hiroyuki Noji, Makoto Kurano

**Affiliations:** a Department of Clinical Laboratory, The University of Tokyo Hospital, Tokyo, Japan; b Department of Applied Chemistry, The University of Tokyo, Tokyo, Japan; c Department of Nephrology and Endocrinology, The University of Tokyo Hospital, Tokyo, Japan

**Keywords:** Blood sampling, Point-of-care testing, Serum extraction, C-reactive protein

## Abstract

**Background:**

The growing demand for point-of-care testing (POCT) has increased interest in at-home blood testing. However, practical methods for obtaining serum from small-volume blood samples remain limited. This study investigated the effects of dilution buffers and additives on RBC aggregation and clot-formation behavior in diluted blood to develop a novel serum extraction method.

**Methods:**

Residual blood samples obtained from outpatients were used to evaluate RBC aggregation and clot-formation behavior with various dilution buffers and additives, including polyethylene glycol (PEG). Based on effective combinations, a filtration-based method for serum extraction from small-volume blood samples was developed and evaluated.

**Results:**

Dilution with saline or phosphate-buffered saline resulted in large clots and numerous floating red blood cells (RBCs). In contrast, the high-molecular-weight PEG reduced clot size and markedly decreased the number of floating RBCs. Hypertonic dilution buffers also reduced floating RBCs without disrupting clot formation. By combining PEG with hypertonic buffers, serum was successfully extracted by manual filtration without hemolysis. The extracted serum enabled C-reactive protein measurement that strongly correlated with measurements obtained from conventional serum samples.

**Conclusions:**

A simple and rapid method for hemolysis-free serum extraction from small-volume blood samples was developed using PEG and hypertonic buffers. This approach may facilitate the development of POCT applications requiring reliable serum collection from limited blood volumes.

## Highlights

• The high-molecular-weight polymer polyethylene glycol reduced clot size.
• Polyethylene glycol and hypertonic buffers reduced floating red blood cells.
• Their combination enabled hemolysis-free serum extraction.
• Extracted serum CRP values strongly correlated with conventional serum.
• The method supports small-volume blood sampling for POCT.

## Abbreviations:

CRP: C-reactive protein
HMW: high-molecular-weight
KPi buffer: potassium phosphate buffer
NaCl: sodium chloride;
NS: normal saline;
PBS: phosphate-buffered saline;
PEG: polyethylene glycol
POCT: point-of-care testing
RBCs: red blood cells
SMBG: self-monitoring of blood glucose

## 1 Introduction

In the current healthcare system, blood tests are essential to managing chronic diseases [1]. As the demand for home health care increases, the need for blood tests, such as point-of-care testing (POCT), is growing rapidly [2]. The RE-ASSURED criteria, originally proposed by the World Health Organization, are widely used to define the optimal characteristics of POCT [3]. It defines the optimal characteristics of POCT tests, including ease of specimen collection, user-friendliness, and being equipment-free.

Currently, with the exception of the self-monitoring of blood glucose (SMBG), few blood tests for POCT are popular at home and have a quantitative performance equal to that of medical facilities. A challenge in the development of a novel blood test for POCT is meeting the 'ease of specimen collection' criterion in the RE-ASSURED criteria. This process is inevitable because, unlike the SMBG test, which can enzymatically or electrically measure the level of blood glucose from whole blood, most of the proteins or enzyme activities from whole blood cannot be measured, as hemoglobin in red blood cells (RBCs) strongly interferes with colorimetric or fluorescent quantification. To solve these problems, several approaches have been investigated, including microfluidic separation [4], filtration of undiluted whole blood [5], and compact or field-deployable centrifugation systems for microvolume blood samples [6]. However, these methods still have limitations in terms of sensitivity, cost, operational simplicity, sample-volume requirements, or device availability.

In the field of blood rheology, RBC aggregates in the presence of high-molecular-weight (HMW) substances by reducing the electrostatic repulsion of erythrocytes [7]. This phenomenon is often observed *in vivo* in the blood of patients with myeloma, where accumulated pathogenic proteins cause rouleaux formation in erythrocytes [7]. The inclusion of polymers, such as HMW dextran or polyethylene glycol (PEG) has also been reported to cause this phenomenon [8].

In this study, we aimed to investigate a novel approach for serum extraction from microblood, inspired by blood rheology. We found that a hypertonic dilution buffer containing an HMW compound synergistically promoted RBC aggregation and compact clot formation, which facilitated serum extraction. Additionally, we demonstrated our new serum extraction method for POCT, which is a readily available and inexpensive method to sort blood cells and obtain serum without costly materials or devices.

## 2 Methods

### 2.1 Ethics

This study was approved by the Ethics Committee of the Graduate School of Medicine and Faculty of Medicine at the University of Tokyo (No. 2021036NI). Informed consent was obtained using an opt-out method, and patients who refused to participate in this study were excluded. All methods were performed in accordance with the relevant guidelines and regulations.

### 2.2 Collection of blood sample

Residual blood samples collected in citrate tubes from outpatients at the University of Tokyo Hospital were used for the coagulation experiments and for serum extraction using the proposed method. The citrate tubes contained liquid sodium citrate anticoagulant at a citrate-to-blood ratio of 1:9, resulting in initial dilution of the blood samples. For comparison of C-reactive protein (CRP) concentrations, matched patient serum samples collected simultaneously in standard serum collection tubes and prepared by centrifugation after clotting were used as reference samples. Blood samples from patients with severe anemia and those receiving antiplatelet or anticoagulant therapy were excluded.

### 2.3 Blood coagulation test

Phosphate-buffered saline (PBS), sodium chloride (NaCl) (FUJIFILM Wako Pure Chemical Corporation, Japan), normal saline (NS, Otsuka Pharmaceutical Factory, Inc., Japan), and potassium phosphate buffer (KPi buffer, Muto Pure Chemical Co. Ltd., Japan) were used to prepare the coagulation solution. PEG (Mw = 200, 2,000, 6,000, 20,000; FUJIFILM Wako Pure Chemical Corporation, Japan) and PEG (Mw = 35,000; Sigma-Aldrich, USA) were used as solutes. Polyoxyethylene polyoxypropylene block polymer pluronic nonionic surfactants (NEWPOL PE-74 [Mw = 3000], PE-75 [Mw = 3500], PE-108 [Mw = 16,000]; Sanyo Chemical, Japan) and dextran (Mw = 60,000 and 200,000; FUJIFILM Wako Pure Chemical Corporation, Japan) were also used as solutes.

In the blood coagulation assay, 50 μL of blood was added to 945 μL of the designated dilution buffer, selected according to the experimental design, and 5 μL of bovine thrombin (0.5 unit/μL; Fuji Pharma Co., Ltd., Japan) in a 5 mL polystyrene tube. The mixture was thoroughly homogenized and incubated for 5 min. Subsequently, the blood sample was transferred to a six-well plate, and the clot morphology was photographed. The number of RBCs suspended in the serum was counted using a Bürker–Türk hemocytometer, and the number of RBCs was evaluated as a relative ratio compared with the number of control samples in each experiment. Images were obtained using an optical microscope (DP73; OLYMPUS Corporation, Japan). All the experiments were conducted at room temperature (18 °C to 25 °C).

### 2.4 Serum extraction from microblood

First, we prepared a commercially available 10-mL dropper bottle containing 945 μL of solution and 5 μL of bovine thrombin (0.5 unit/μL, Fuji Pharma Co., Ltd., Japan). Subsequently, 50 μL of blood was added to the dropper bottle by micropipette. After mixing, the samples were incubated for 5 min at room temperature. After incubation, the dropper was connected to a syringe filter (cellulose acetate membrane: pore size, 5 μm; diameter, 25 mm; GVS North America, USA) upside down. Filtration was performed manually, and the dropper was squeezed until the solution in the dropper was empty. The extracted serum was collected in microtubes and photographed, and the degree of hemolysis was evaluated by measuring the absorbance at 414 nm using a spectrophotometer (NanoDrop One, Thermo Fisher Scientific, USA).

### 2.5 Measurement of C-reactive protein (CRP) concentration in serum

For C-reactive protein (CRP) concentration analysis, serum was extracted from 50 μL of citrated residual blood using the proposed method described above. CRP concentrations in the extracted serum were measured in the ISO 15189-certified clinical laboratory of the University of Tokyo Hospital. The extracted serum was analyzed using the LZ test Eiken CRP-HG (Eiken Chemical Co., Ltd., Japan), based on a latex agglutination turbidimetric immunoassay. The measured CRP concentrations were compared with those obtained from matched patient serum samples collected simultaneously in standard serum collection tubes and prepared by centrifugation after clotting. The same reagents were used for both samples. Because the citrate tubes contained liquid sodium citrate anticoagulant at a citrate-to-blood ratio of 1:9 under the experimental conditions of this study, and because the proposed method further diluted the original blood sample during extraction, the CRP concentrations measured in the extracted serum were not corrected for dilution.

### 2.6 Statistical analyses

Data processing and analysis were performed using the GraphPad Prism 9.5.1 software (GraphPad Software, San Diego, CA, USA). Statistical comparisons among multiple groups were performed using one-way analysis of variance followed by Dunnett's multiple comparison test or Tukey's multiple comparison test, as appropriate. The specific post hoc test used for each experiment is indicated in the corresponding figure legend. For comparison of CRP concentrations between the extracted serum and matched conventional serum samples, simple linear regression analysis was performed, and the coefficient of determination (R^2^) and *P*-value for the regression were calculated. Results were considered significant when the *P*-value was <0.05. The *P*-values were described as * for <0.05, ** for <0.01, *** for <0.001, and **** for <0.0001.

## 3 Results

### 3.1 Addition of high-molecular-weight (HMW) compounds improves thrombin-induced clot formation and reduces floating RBCs in diluted small-volume blood samples

**Fig. 1:**
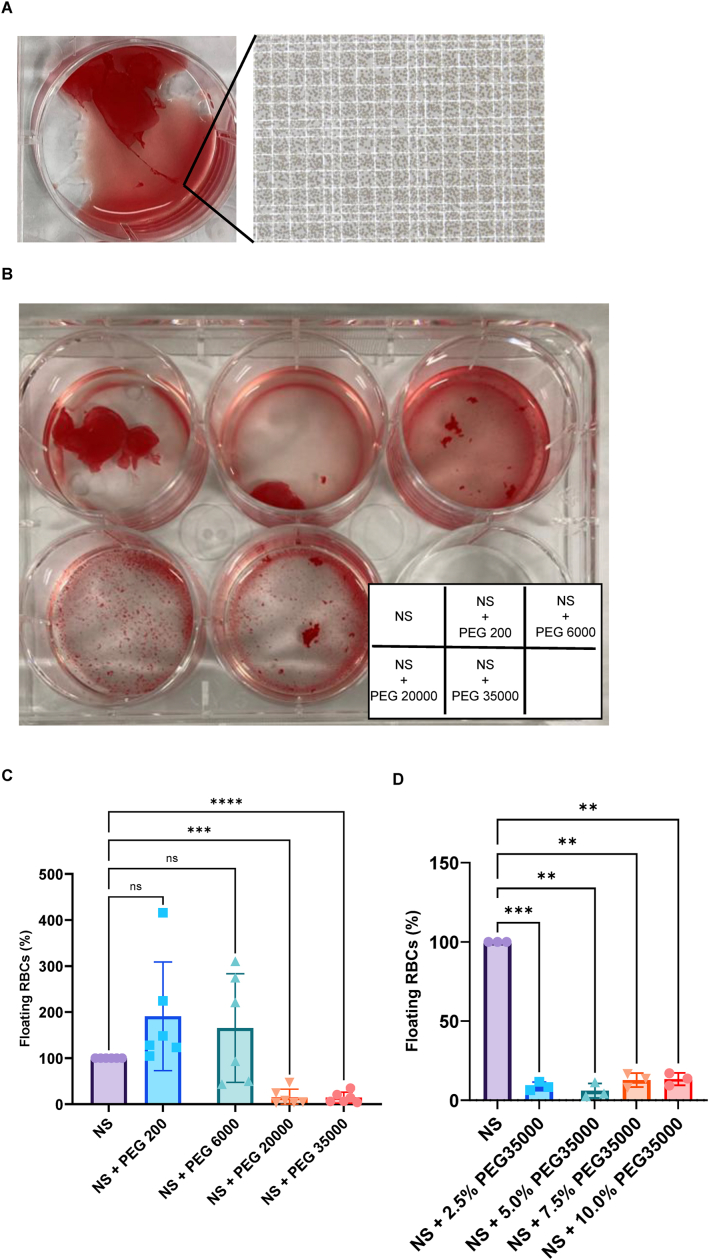
High-molecular-weight polyethylene glycols (PEGs) in dilution buffer improve thrombin-induced clot formation and reduce floating RBCs (A) (left panel) Macroscopic image of clot of blood diluted with normal saline (NS). (right panel) Microscopic image of floating red blood cells (RBCs) in serum of NS-diluted blood in Bürker–Türk counting chamber. (B) Macroscopic images of clots of blood diluted with NS, containing varying molecular weights of PEG at a concentration of 5% w/v. (C) The relative numbers of floating RBCs in serum from blood diluted with NS, containing varying molecular weights of PEGs at a concentration of 5% w/v. (D) The relative numbers of floating RBCs in serum from blood diluted with NS containing varying concentrations of PEG (Mw = 35000). Data are represented as the means ± SDs (n = 3–6). The number of floating RBCs in each sample was normalized to that of the control sample (NS). The *P*-values are denoted as * <0.05, ** <0.01, *** <0.001, and **** <0.0001 (Dunnett's test). SDs, standard deviations.

To perform certain blood tests for POCT using microblood, which is usually limited to <60 μL, dilution is often necessary to obtain a sufficient sample volume. Initially, we analyzed thrombin-induced clot formation in diluted blood because residual RBCs interfere with clinical chemistry testing. As shown in Fig. 1A, NS-diluted blood formed large clots after thrombin addition. Moreover, the resulting serum contained numerous floating RBCs, indicating inefficient RBC incorporation into the thrombin-induced clot. Both these features are considered unfavorable for testing serum samples; therefore, we studied a method to improve clot-formation behavior and reduce floating RBCs.

We then tested whether the addition of PEG of different molecular weights to NS would modify RBC aggregation and clot-formation behavior, based on insights from rheology. Notably, the addition of HMW (Mw > 10,000) PEGs (5% w/v) suppressed the formation of large blood clots, which was not observed with the addition of low-molecular-weight (Mw < 10,000) PEGs (Fig. 1B). Moreover, the number of floating RBCs in the extracted serum was significantly reduced by the addition of HMW PEGs (Fig. 1C). We also tested the optimal concentration of HMW PEG for reducing floating RBCs during clot formation and found that approximately 5% w/v final concentration of HMW PEG was the most efficient (Fig. 1D).

**Fig. 2:**
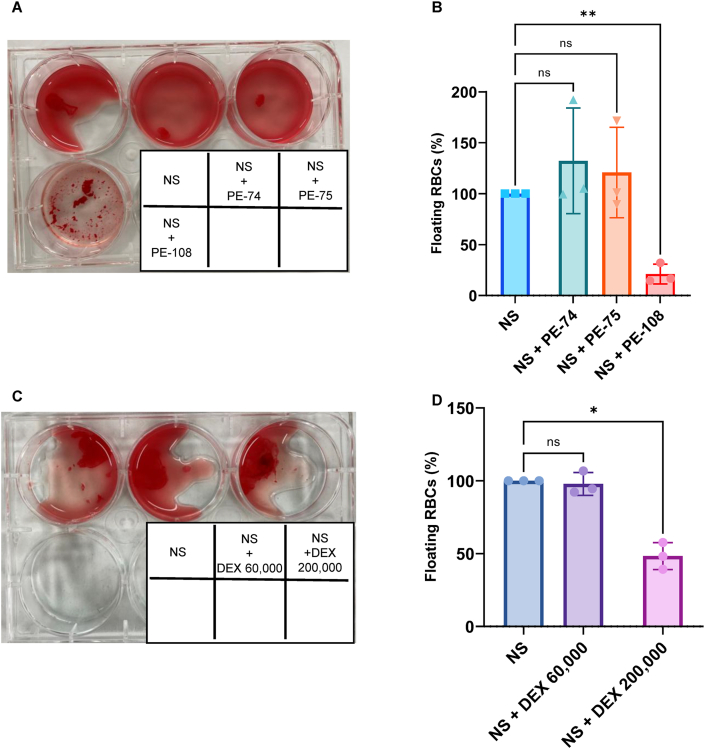
High-molecular-weight compounds in solution alter clot-formation behavior and reduce floating RBCs (A) Macroscopic images of clots of blood diluted with normal saline (NS) containing various types of polyoxyethylene polyoxypropylene block polymer pluronic nonionic surfactants. PE-74: Mw = 3000, PE-75: Mw = 3500, PE-108: Mw = 16000 (B) The relative numbers of floating red blood cells (RBCs) in serum from blood diluted with NS containing varying molecular weights of polyoxyethylene polyoxypropylene block polymer pluronic nonionic surfactants. (C) Macroscopic images of clots of blood diluted with NS, containing dextran (DEX) of two different molecular weights. (D) Relative numbers of floating red blood cells in serum from blood diluted with NS, containing two molecular weights of DEX. Data are represented as means ± SDs (n = 3). The number of floating RBCs in each sample was normalized to that of the control sample (NS). The *P*-values are denoted as * <0.05, ** <0.01 (Dunnett's test). SDs, standard deviations.

We also tested another type of HMW compound, polyoxyethylene polyoxypropylene block polymer pluronic nonionic surfactant and dextran with different molecular weights (Fig. 2A–D). Notably, although the HMW polyoxyethylene polyoxypropylene block polymer pluronic nonionic surfactant suppressed the formation of large blood clots and reduced floating RBCs, similar to PEGs (Fig. 2A and B), HMW dextran showed significantly less of an effect than PEGs on both clot formation and reducing floating RBCs (Fig. 2C and D).

### 3.2 Hypertonic dilution buffers reduce floating RBCs during thrombin-induced clot formation

**Fig. 3:**
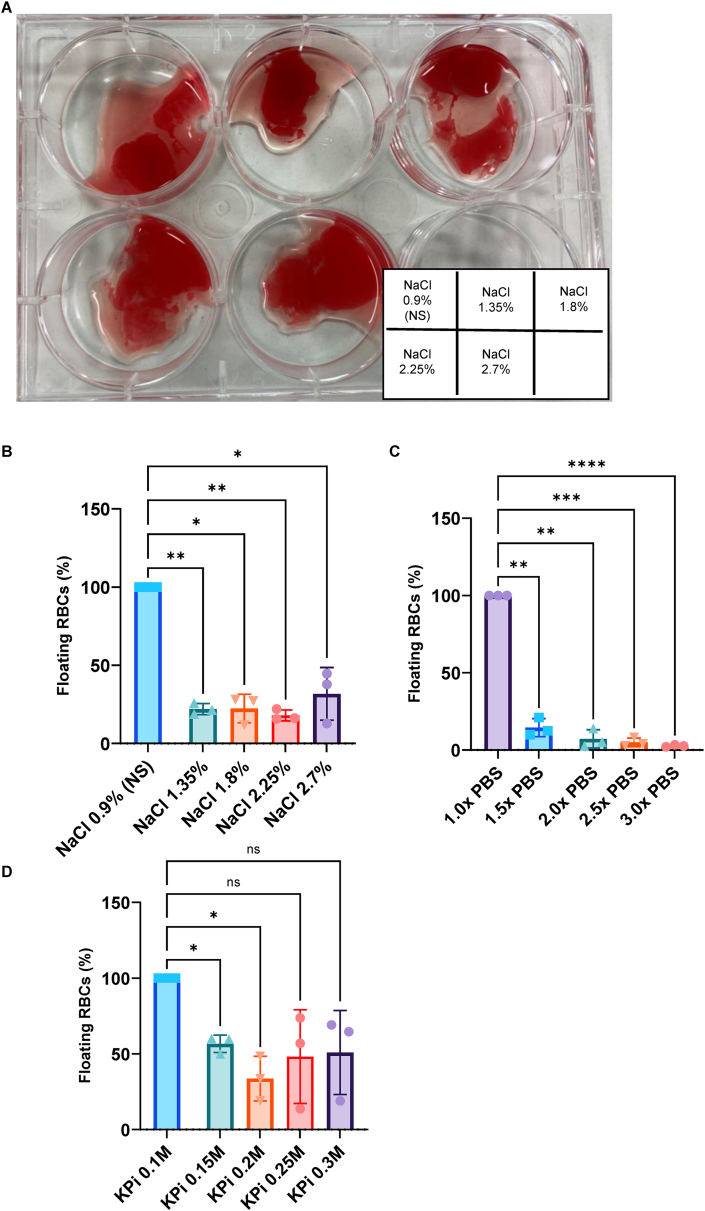
Hypertonic dilution buffers reduce floating RBCs during thrombin-induced clot formation (A) Macroscopic image of clots of blood diluted with various concentrations of sodium chloride (NaCl) solution. (B) Relative numbers of floating red blood cells (RBCs) in serum from blood diluted with various concentrations of NaCl solution. (C) Relative numbers of floating RBCs in serum from blood diluted with various concentrations of phosphate-buffered saline. (D) Relative numbers of floating RBCs in serum from blood diluted with various concentrations of potassium phosphate buffer. Data are represented as means ± SDs (n = 3). The *P*-values are denoted as * <0.05, ** <0.01, *** <0.001, and **** <0.0001 (Dunnett's test). SDs, standard deviations.

To investigate the effects of a hypertonic environment on RBC aggregation and clotting, we examined whether the properties of the dilution buffer affected clot formation and the number of floating RBCs. In the clot-formation experiment using hypertonic saline, the macroscopic size of the produced blood clots remained unchanged (Fig. 3A). However, the number of floating RBCs in the serum was significantly lower in the hypertonic dilution buffer (Fig. 3B). The number of floating RBCs was minimized at an approximately 1.8% (w/v) NaCl concentration in the dilution buffer, which was twice the osmotic pressure of NS. The same trend was observed in PBS and sodium ion-free KPi buffer (Fig. 3C and D). Based on these experimental results, we concluded that the hypertonic dilution buffer does not affect clot formation, but reduces floating RBCs.

### 3.3 Combination of HMW polyethylene glycol (PEG) and hypertonic dilution buffer markedly promotes RBC aggregation and improves clot morphology

**Fig. 4:**
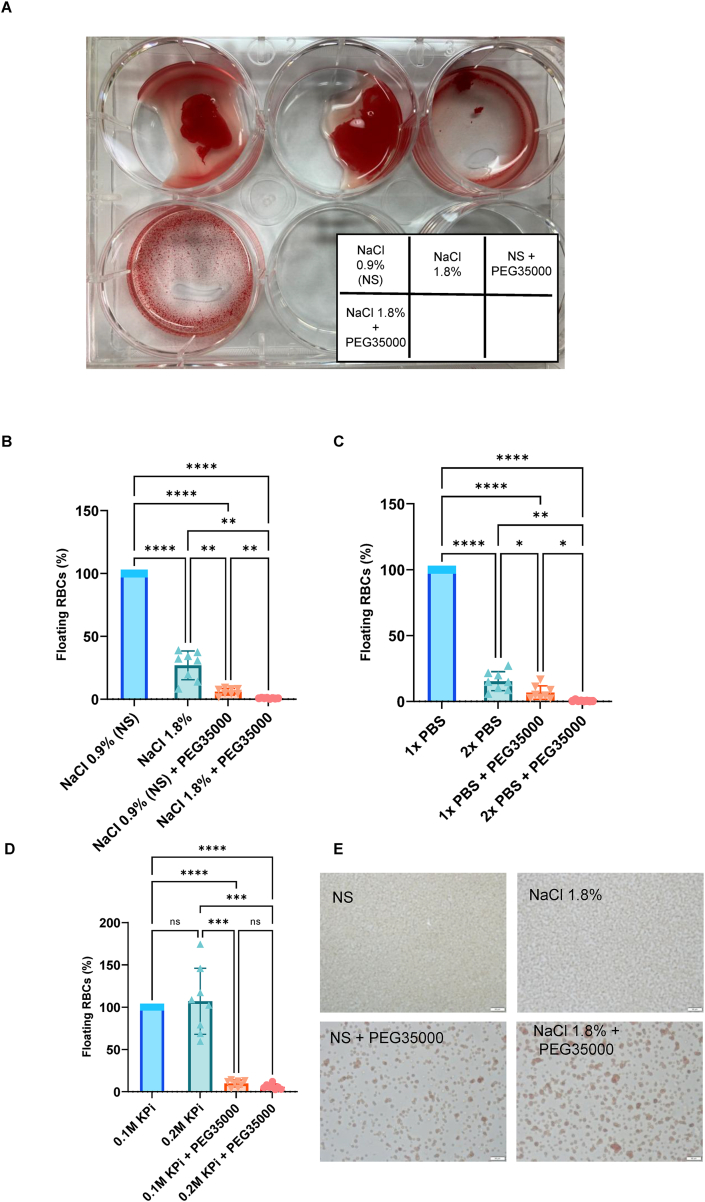
Combination of high-molecular-weight polymer and hypertonic dilution buffer promotes RBC aggregation and improves clot morphology (A) Macroscopic images of blood clots diluted with a high-molecular-weight polymer in isotonic or hypertonic saline. (B) Relative numbers of floating red blood cells (RBCs) in the serum from blood diluted with a combination of high-molecular-weight polyethylene glycol in either isotonic or hypertonic saline. (C) Relative numbers of floating RBCs in serum from blood diluted with a combination of high-molecular-weight polyethylene glycol in either isotonic or hypertonic phosphate-buffered saline. (D) Relative numbers of floating RBCs in the blood serum from blood diluted with a combination of high-molecular-weight polyethylene glycol in either isotonic or hypertonic potassium phosphate buffer. (E) Microscopic images showing erythrocyte aggregation in blood diluted with a high-molecular-weight polyethylene glycol in either isotonic or hypertonic saline. Data are represented as means ± SDs (n = 8). The *P*-values are denoted as * <0.05, ** <0.01, *** <0.001, and **** <0.0001 (Tukey's test). SDs, standard deviations.

Based on the results obtained thus far, we hypothesized that the combination of HMW PEG and hypertonic dilution buffer could promote RBC aggregation, improve clot-formation behavior in diluted small-volume blood samples, and minimize residual floating RBCs. As shown in Fig. 4A and B, the combination of HMW PEGs and hypertonic saline significantly suppressed the formation of large blood clots, and the number of floating RBCs contained in the extracted serum was significantly lower compared with either one alone. This tendency was reproduced in other dilution buffers, PBS and KPi (Fig. 4C and D). We also found that the RBCs in the diluted blood strongly aggregated (erythrocyte aggregation) in a solvent consisting of HMW PEG and hypertonic saline without thrombin (Fig. 4E). Based on these findings, we speculate that the combination of HMW PEG and hypertonic dilution buffer accelerates RBC aggregation, thereby promoting the formation of compact clots and reducing residual floating RBCs.

### 3.4 Novel serum extraction method based on HMW PEG- and hypertonic buffer-induced RBC aggregation and compact clot formation

**Fig. 5:**
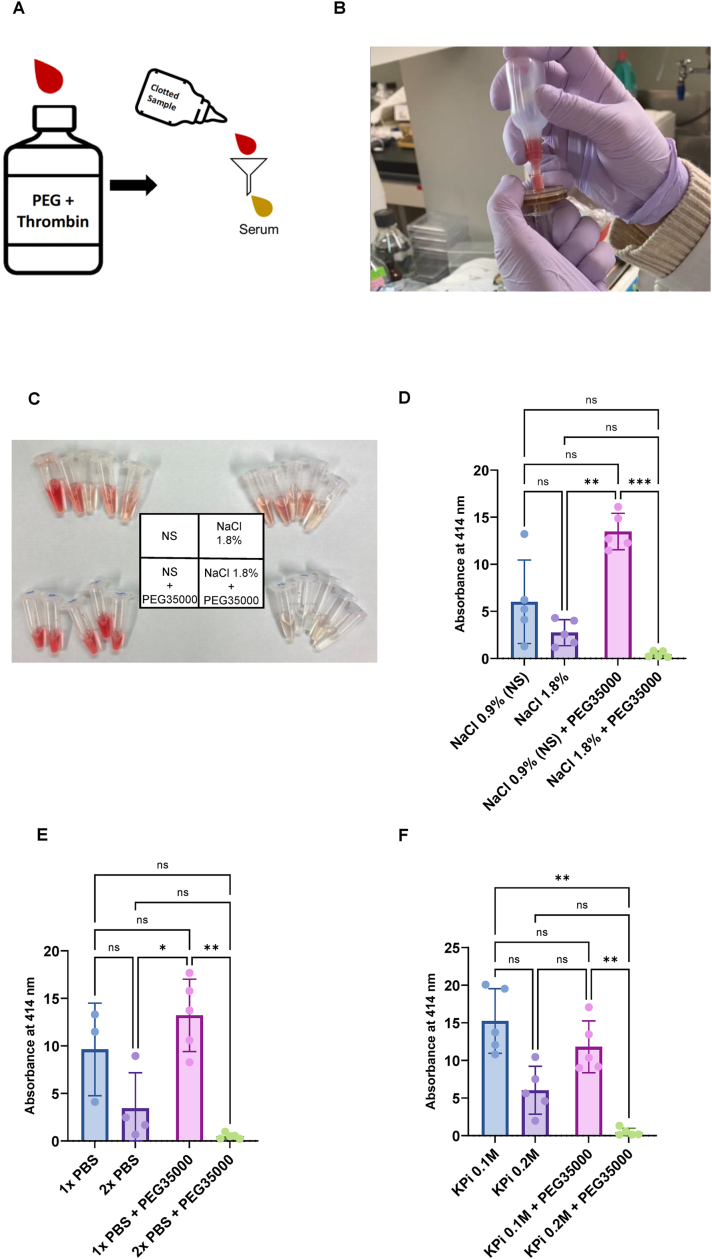
Novel method for serum extraction from small-volume blood samples (A) Schematic illustration of a novel serum extraction method from a microblood sample. (B) Photograph of the serum extraction procedure. (C) Results of serum extraction from blood diluted with a combination of 5% w/v polyethylene glycol (PEG) (molecular weight 35,000) in either isotonic or hypertonic saline. (D–F) Absorbance at 414 nm indicating the degree of hemolysis during serum extraction using either isotonic or hypertonic solutions of sodium chloride (D), phosphate-buffered saline (E), or potassium phosphate (F) as buffers, with or without 5% w/v PEG. Data are represented as means ± SDs (n = 5). The *P*-values are denoted as * <0.05, ** <0.01, *** <0.001, and **** <0.0001 (Tukey's test). SDs, standard deviations.

Following the identification of factors that promote RBC aggregation and compact clot formation in diluted blood, we utilized this finding to attempt the development of a novel serum extraction method from microblood. A schematic representation of the proposed method is shown in Fig. 5A. We developed a prototype of this serum extraction method using a commercially available 10-mL dropper bottle and a syringe filter with a 5-μm pore-size membrane (Fig. 5B).

Blood diluted with NS underwent hemolysis by filtering, and neither the addition of HMW PEG nor hypertonic saline alone successfully extracted serum without hemolysis. However, the combination of HMW PEG and hypertonic saline enabled serum extraction without hemolysis (Fig. 5C and D). This phenomenon was also observed in PBS and KPi buffer (Fig. 5E and F). Based on these results, we conclude that the combination of HMW PEG and a hypertonic dilution buffer enables serum extraction with reduced hemolysis compared with cases without this combination.

### 3.5 Extracted serum showed a strong correlation with conventional serum in CRP analysis

**Fig. 6:**
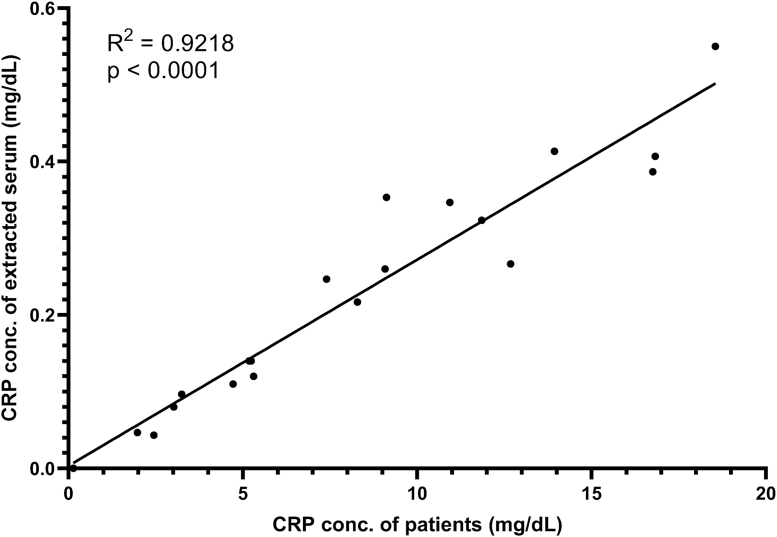
Correlation of C-reactive protein (CRP) concentrations measured in serum extracted using the novel method The x-axis represents the CRP concentration in the patient's serum extracted using the conventional method, whereas the y-axis represents the CRP concentration in the serum extracted using the novel method. Twenty specimens were analyzed; some data points overlap. A linear regression line is shown with a coefficient of determination R^2^ = 0.9218.

To evaluate whether the serum obtained using our novel method could be used for immunological analysis, we compared CRP concentrations measured in serum extracted using the proposed method with those measured in matched conventional serum samples. As shown in Fig. 6, a strong positive linear correlation was observed between the CRP values measured in serum extracted using the proposed method and those measured in matched conventional serum samples (R^2^ = 0.9218, P < 0.0001) (Fig. 6). These results suggest that the serum extracted using our novel method is compatible with CRP measurement using immunological assays, although the measured values were not corrected for dilution.

## 4 Discussion

In this study, we demonstrated that a combination of HMW PEGs and hypertonic dilution buffer promoted RBC aggregation and altered clot-formation behavior in small-volume blood samples. They suppressed the formation of large clots and significantly reduced the number of floating RBCs in the extracted sera. These aggregation and clot-formation characteristics enabled the development of a simple and straightforward serum extraction method. We also demonstrated that the extracted serum was suitable for immunological assays. To the best of our knowledge, this is the first study demonstrating a specific procedure for obtaining serum from microblood samples.

As the demand for POCT is increasing, the need for blood tests using microblood is also increasing. However, a method for removing blood cells remains an unsolved issue. Unlike previous approaches, we conceived a novel method that promotes RBC aggregation and compact clot formation using HMW PEG and a hypertonic dilution buffer, which enables serum extraction by simple filtration.

Throughout our experiments, we demonstrated that the addition of HMW PEGs to the dilution buffer dramatically enhanced erythrocyte aggregation and promoted the formation of small, compact blood clots during thrombin-induced clot formation. As shown in Fig. 4E, the presence of HMW PEG in the dilution buffer strongly enhanced erythrocyte aggregation before thrombin-induced clot formation. We hypothesized that this aggregation of RBCs leads to the formation of more stable and smaller blood clots and reduces free RBCs during the clot-formation process.

In the field of blood rheology, natural or synthetic HMW compounds can induce RBC aggregation in the bloodstream [7,8]; however, the effect of adding a high volume of HMW PEGs, which are toxic to living organisms [9], on RBC aggregation and clot formation is less studied because it is highly unlikely to occur in the human body. However, in clinical testing, the toxicity of the reagents to the organism is less of a concern, allowing the use of this phenomenon.

In addition to HMW PEGs, we showed that a hypertonic dilution buffer promoted RBC aggregation and reduced residual floating RBCs. We assumed that this phenomenon could be explained by erythrocyte aggregation. Erythrocyte aggregation is also induced by certain physicochemical conditions; one condition is induction in a hypertonic buffer [10]. The observation that this phenomenon is confirmed not only in hypertonic saline but also in hypertonic PBS or potassium-based buffers supports the idea that osmotic pressure, not the type of ion, is essential for this phenomenon.

Based on our findings, we developed serum extraction methods for microblood. Unlike dilution with NS, the combination of HMW PEGs and hypertonic dilution buffer promoted RBC aggregation, restrained the formation of large blood clots, and dramatically reduced the number of remaining free erythrocytes in the serum. This made it possible to extract serum through simple filtration without occlusion of the circuit caused by blood clots or hemolysis. We also showed that the serum extracted using this method was compatible with immunological CRP measurement and showed a strong correlation with matched conventional serum samples.

Our method possesses some optimal features of the RE-ASSURED criteria: ease of specimen collection, affordability, user-friendliness, rapid and robust, equipment-free, and deliverable to end users [3]. Given these characteristics, we believe that this method will contribute to the advancement of blood POCT.

This study has some limitations. First, the proposed method requires dilution of the original blood sample during the extraction step to obtain a sufficient volume of extracted serum for downstream analysis. Therefore, the serum obtained using this method represents a diluted serum fraction. Optimization of the extraction volume, dilution conditions, and correction strategy will be necessary according to the target analyte and assay method.

Second, this study used blood samples stored in a clinical laboratory setting, and whether serum can be successfully extracted from fresh patient blood samples remains unclear. For implementation in clinical practice, further refinement of the extraction device and feasibility testing using real patient samples are required.

Third, the effect of hematocrit variability was not fully evaluated in this study. Because the actual serum or plasma volume contained in 50 μL of whole blood decreases as hematocrit increases, interindividual differences in hematocrit may affect the effective dilution factor. This may partly account for the deviation of the regression line in Fig. 6 from perfect agreement, despite the strong correlation between measurements obtained using the proposed method and those obtained using conventional samples. Although measurement of an internal standard could help correct for hematocrit-dependent variation, incorporating such an additional measurement may increase the complexity and cost of the device, which would be disadvantageous for point-of-care testing applications. Therefore, the need for hematocrit correction should be evaluated according to the intended analyte and device configuration in future studies.

Fourth, the serum extracted in this study was hypertonic and contained PEG, which may make it unsuitable for direct use in enzyme activity assays. However, considering that PEG can be replaced with other HMW molecules and that specific compositions are not required for hypertonicity, based on the basic concept of this study, we believe that a more suitable sampling system for enzymatic methods can be developed. There is a strong clinical demand for enzyme activity assays; therefore, it would be meaningful to explore solvent and solute conditions or incorporate additional processing steps to enable such assays in the future.

In conclusion, we demonstrated a novel sampling strategy for at-home blood testing based on the need for rapid, hemolysis-free serum extraction based on blood rheology. We hope that our discoveries and methods will contribute to the improvement of at-home blood testing and will be beneficial for health management.

## Research funding

This research was supported by 10.13039/100009619AMED under Grant Number JP24zf0127006h0003. The funder had no role in the study design, data collection, analysis, interpretation, writing of the manuscript, or decision to submit the article for publication.

## CRediT authorship contribution statement

**Hideaki Isago:** Conceptualization, Formal analysis, Investigation, Methodology, Resources, Writing – original draft, Writing – review & editing. **Baasanjav Uranbileg:** Investigation, Resources, Writing – review & editing. **Keisuke Murayama:** Investigation, Resources, Writing – review & editing. **Yuki Nakano:** Investigation, Resources, Writing – review & editing. **Yukio Kume:** Investigation, Resources, Writing – review & editing. **Yoshihiro Minagawa:** Investigation, Resources, Writing – review & editing. **Masaomi Nangaku:** Conceptualization, Funding acquisition, Project administration, Supervision, Writing – review & editing. **Hiroyuki Noji:** Conceptualization, Funding acquisition, Supervision, Writing – review & editing. **Makoto Kurano:** Conceptualization, Funding acquisition, Investigation, Methodology, Project administration, Resources, Supervision, Writing – review & editing.

## Declaration of competing interest

The authors declare the following financial interests/personal relationships which may be considered as potential competing interests:Hideaki Isago, Yoshihiro Minagawa, Masaomi Nangaku, Hiroyuki Noji, and Makoto Kurano have patent pending to The University of Tokyo. If there are other authors, they declare that they have no known competing financial interests or personal relationships that could have appeared to influence the work reported in this paper.

## Data Availability

Data will be made available on request.
